# Nanoceutical Adjuvants as Wound Healing Material: Precepts and Prospects

**DOI:** 10.3390/ijms22094748

**Published:** 2021-04-29

**Authors:** Kaushita Banerjee, Radha Madhyastha, Yuichi Nakajima, Masugi Maruyama, Harishkumar Madhyastha

**Affiliations:** 1 Department of Biomedical Sciences, School of Biosciences and Technology, Vellore Institute of Technology, Vellore 632014, India; kbgee99@gmail.com; 2 Department of Applied Physiology, Faculty of Medicine, University of Miyazaki, Miyazaki 8891692, Japan; radharao@med.miyazaki-u.ac.jp (R.M.); yunakaji@med.miyazaki-u.ac.jp (Y.N.); masugi@med.miyazaki-u.ac.jp (M.M.)

**Keywords:** dermal wound healing, nanoceuticals, metal nanoparticles, bioengineered alternatives

## Abstract

Dermal wound healing describes the progressive repair and recalcitrant mechanism of 12 damaged skin, and eventually, reformatting and reshaping the skin. Many probiotics, nutritional supplements, metal nanoparticles, composites, skin constructs, polymers, and so forth have been associated with the improved healing process of wounds. The exact mechanism of material-cellular interaction is a point of immense importance, particularly in pathological conditions such as diabetes. Bioengineered alternative agents will likely continue to dominate the outpatient and perioperative management of chronic, recalcitrant wounds as new products continue to cut costs and improve the wound healing process. This review article provides an update on the various remedies with confirmed wound healing activities of metal-based nanoceutical adjuvanted agents and also other nano-based counterparts from previous experiments conducted by various researchers.

## 1. Introduction

Human tissue skin consists of stratum corneum, an intricate mechanical boundary that distinguishes and safeguards internal body structures from external responses. A typical stratiform epithelium is alienated into the overlying epidermal layer with keratinocytes enclosed by the basal membrane, melanocytes, and Langerhans and Merkel cells, as well as the dermis with fibroblasts and a collagen-rich extracellular matrix and elastic fibers along with blood and lymph vessels, sebaceous glands, nerve endings, and the underlying hypodermal layer. As the skin is continually subjected to many external factors, it is highly vulnerable to wound injury or trauma [1]. Wounds or abrasions impair the stratum corneum’s anatomic structure, causing a breakdown of the surface and other soft tissues, thus altering its normal function. A wound can be a cut, scratch, scrape, punctured skin, hematoma, contusion, avulsions, etc. as an outcome of the physio-pathological process that can occur to any organ by external or internal forces [1]. Wounds disintegrate the local environment within and around the tissue leading to hemorrhage, vasoconstriction, clotting, complement activation, and pre/post-inflammatory responses. Wound healing is an active, intricate, and complex process. Commencing from wound formation, it encompasses several soluble mediators and extracellular matrices along with fibroblast accumulation, epithelial cell migration, replication, and reorganization of tissues, finally leading to the reparation of its anatomic and functional integrity to establish homeostasis [2,3]. Damage to the tissue is inevitable and may extend from a minor scrape to a complex and intricate impairment. During normal tissue repair, the interaction between platelets, fibroblasts, endothelial cells, keratinocytes, and extracellular matrix (ECM) components is regulated by multiple biochemical moieties, cytokines, and growth factors to initiate the refurbishment of the damaged dermal layer [3]. Although the process of tissue damage to repair follows an unhindered continuum, it could sometimes be flawed due to diverse factors following an anomalous healing trajectory.

Wounds can be of different categories depending on their etiology, site, causative agent, complexity, infliction, treatment, and curative period [4]. A simple bruise or contusion results from a blood vessel rupture and appears as black-blue marks on the skin surface. Avulsion, laceration, cuts, abrasions, punctures, bite, and burns are primary open traumatic wounds limited to the cutaneous and subcutaneous layers of the stratum corneum and its intrinsic tissues. Acute traumatic wounds occur when the skin’s epidermis/dermis layer is ruptured with a penetrating injury [5,6]. Wounds also differ in their pathophysiology and medical supervision. A bite could be a clean cut in semblance, but prone to contamination, thus requiring extra medical management [7]. Burn wounds are characterized by excessive blood loss and augmented capillary penetrability, leading to bloodstream infection in patients with an impaired immune system. Deep burns show delayed re-epithelialization and restoration and are the most prone to bacterial infection. Based on the extent of injury, burn wounds can be classified as first-, second-, or third-degree injuries [8,9].

Categorically cutaneous wounds are widely classified either as acute or chronic. “Acute wounds” are restored via a chronological course of healing, viz., inflammation, tissue development, and restoration occurring in a particular orderly fashion. The healing encompasses a complex sequential collective of cell motility and differentiation, new blood vessel formation, and the structural development of ECM along with scar tissue restoration, modulated by several key mediators like thrombocyte, cell signal-mediating glycoproteins, numerous inflammatory cells, matrixins, etc. Such wounds have distinct overlying hemostatic, proliferative, and maturation phases, with an avascular scar ultimately. Injuries caused by accidents, trauma, burns, and surgical procedures give rise to acute wounds where the trajectory of wound contraction and tissue re-epithelialization is precisely time dependent [10,11]. However, a lengthy or delayed restorative trajectory may lead to the formation of “chronic wounds”. Such wounds are often a challenge for treatment, having almost no orderly healing and poor tissue repair. Such recurrent, protracted wounds also result in weakened tissue restoration, and are linked to abnormal anatomical or physiological conditions, on example being the diabetic foot ulcers where marginal neuropathy and subsequent anomalies give rise to viable and permeable tissues with dysregulated and continual inflammation, sometimes leading to unwarranted deposition of collagen and development of an abnormal scar [12,13]. Handling chronic wounds also leads to a significant capital crunch in the healthcare sector. Non-healing wounds are subjected to an increased proteolytic and metalloproteinase profile, which further makes the healing difficult, unlike the acute ones, where a proper balance between the growth factors and cellular responses is mediated [14]. Surgical wounds could result from any incision, excision, or debridement during surgery. Such surgical site infections differ in their size and healing time depending on the extent of wound depth, and usually occur within a month of postoperative events. Infections can also give rise to wounds that need prolonged medicaments for their curing [15], such as in diabetic foot ulcers. These infectious neuropathic wounds can progress to sepsis, followed by gangrene and fluctuating blood glucose levels in patients. Decubitus ulcers or bedsores are wounds in the epidermis/dermis layer of the body areas subjected to continued and relentless pressure, such as the tailbone, hips, elbows, ankles, etc. Osteomyelitis and osteoradionecrosis are bone infections that traverse the bloodstream, infecting the nearby tissue exposed to the surroundings, or can be an effect of prolonged doses of radiation cutting off the blood supply at that particular region, like in the mandibular bone [16].

**Figure 1:**
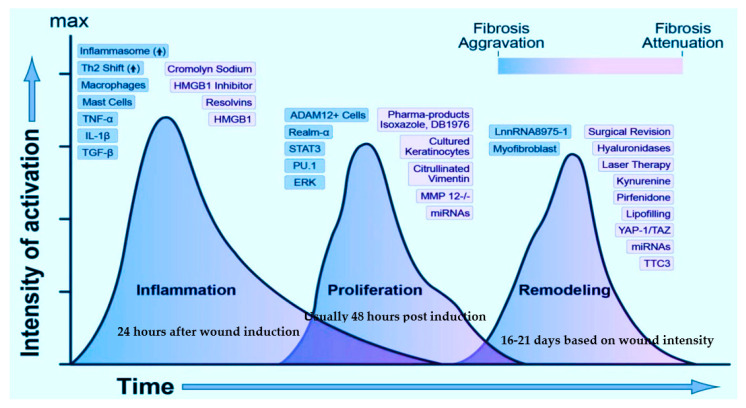
Diagrammatic representation of the overlapping phases of wound healing. Several molecular, biomechanical, and biochemical intermediaries that regulate the healing process are depicted here. The blue section in the figure shows activation, and pink shows diminution of fibrosis. (Adapted from reference [18] respectively, open access)

Regeneration of new tissues post-wounding happens in an intricate non-linear fashion with cell-specific growth and inflammatory factors triggering phlogistic jamboree, with the inflamed migratory cells and cytokines being transported in and around the wound site along with ECM and collagen accumulation and scarring. The synergistic interaction involves cell–cell communication mediators, including peptide molecules, eicosanoids, protein-associated molecular pattern receptors, exosomes, and non-coding ribonucleic acids (ncRNAs). [17]. The healing process tangentially traverses three major phases viz., inflammation, proliferation, and remodeling. During the process, cellular interactions lead to neovascularization, collagen synthesis, re-epithelialization, and wound closure (Figure 1). Variances in the degree of wound reparation are a consequence of the tissue type and injury extent. Partial-thickness wounds require new epithelial regeneration with nominal connective tissue formation. On the other hand, full-thickness wounds require the synthesis of new blood vessels, collagen, glycoproteins, proteoglycans, and epithelial cells for complete healing [18,19].

Under normal conditions, substantial healing over a stipulated time period results in a complete restoration of the epidermal barrier and a partial restoration of the deep dermis, with some scarring and tissue loss. Molecular and cellular effects form the backbone for advanced healing. The activation of inflammatory and migratory cells represents the primary healing phase during which hemostatic balance is restored. In the transitional and granulation phase, keratinocytes and fibroblasts proliferate, migrate to the wound site, and deposit the matrix, refurbishing the ECM. New blood vessels also sprout from pre-existing ones. Chemokines, cytokines, and growth factors also contribute to wound closure and epidermal restoration. Nonetheless, there are usually two possibilities with an amiss restorative response: a chronic wound or a hypertrophic scar [20,21]. The molecular pathobiological mechanisms are weakened in non-healing wounds, culminating in hyperproliferation, abnormal cellular infiltration, development of polymicrobial biofilms, and further infection. Chronic wounds are also subjected to fibroblast cells’ senescence, a shortage of stem cell activation, and hindrances in ECM remodeling [22]. Temporally unhealed wounds can upsurge the possibilities of vascular inadequacy and a high risk of chronic mechanical stress and other comorbidities.

Wound care and management is an age-old practice, dating back from ‘Egyptian papyri’ to the Crimea battlefields, where healing was accomplished by bandage dressings of honey, grease, and lint to curb the secondary infection [23]. Presently, there are great advancements in wound care, and much more scientifically and industrially feasible progress is in the picture. The initial step of any wound treatment is its bed preparation, once the underlying cause of the wound has been addressed. Wound bed preparation is done to optimize the healing process in which the wound is cleaned and made devoid of any debridement (nonviable devitalized wound tissue) to obtain a healthy granulation tissue section. Removal of devitalized tissue can be achieved using mechanical, surgical, enzymatic, and autolytic methods [22].

The most traditional approach for wound healing is the use of dressings. A dressing should be moist for ready absorption of exudate and maintain a moisture balance within the wound’s exterior [24]. The most conventional dressings were the wet-to-dry gauze type, where decreased re-epithelialization and a dry gauze surface caused deprived healing and tissue impairment. Then came moist dressings, or ‘occlusive’ dressings, as they are widely known. An optimal moisture balance encourages cell proliferation, averts inflammation, balances the oxygen tension, and optimizes exudation in/around the wound. It further enables autolytic debridement, thus accelerating healing minus the chances of substantial infections [25]. Clinically proven, moisture-retentive dressings have high moisture vapor transmission rates that allow for a timely recovery [26]. Studies by Kannon and Garrett [26] have shown the clinical efficacy of moisture-retentive dressing materials in non-healing wounds. Cordts et al. [27] discussed the efficacy of cost- and time-compliant hydroactive dressings in treating venous leg ulcers. Films or foams of hydrocolloid materials are currently used in moisture-retentive dressings [24]. Polyurethane sheets are also hydrocolloid-amenable dressing materials with strong adhesion onto the wound matrix and promoting autolytic devitalization of the nonviable tissues in contact with exudates. The colloidal counterpart balances the moisture vapor transmission within the wounds. Several scientific studies have shown improved barrier repair using hydrocolloids [28]. Adsorbent wound dressings with alginic acid and colloidal gels such as constituents maintain the hemostatic and fluid equilibrium through calcium-sodium interchange in the system. Their three-dimensional cross-linking polymeric bonds in the liquid gels offer additional precedence for dry necrotic wound beds [28]. Vacuum-aided negative pressure therapy is a crucial technology compatible for diabetic, pressure ulcers, traumatic, surgical wounds, skin grafts, etc. It is supposed that this therapy speeds up wound repair by sustaining moisture around the wound edge, reducing edema, and stimulating angiogenesis and granulation tissue deposition. In a randomized controlled experimental analysis, Soares and coworkers [29] found that negative pressure therapy facilitated bacterial load reduction and quickened wound contraction, unlike the conventional moist gauze dressings. Biologically engineered skin equivalents mimic the stratum corneum structure and trigger several tandem reactions to replicate healing as it happens in a natural skin surface. These dermo-epidermal combination skin constructs are highly effective in treating diabetic and venous ulcers. Topical adjuvants, autologous skin grafting, and hyperbaric oxygen treatments have also been explored for disease-specific management of chronic wounds. However, the lack of clinical data on biological mechanisms has left ambiguity in the treatment efficacy, and further experimental evidence is a prerequisite for its wide usage.

Therefore, it is crucial to develop clinically relevant bioengineered alternative agents to contemplate the process of tissue repair and refurbishment. These adapt to principles of design and analysis to biological systems and biomedical technologies. Bioengineering is applying engineering practices in the fields of medicine and biology. Bioengineering alternative agents could be any form of biologically engineered materials, instrumentation or cell substitutes, approaches, etc., that can branch into medical, agricultural, environmental, bionics, human-machine, biochemical, and genetic engineering that can be implemented for an array of end applications—ranging from medical instrumentation and medical electronics to major areas of interests not restricted to biological modeling, blood flow dynamics, prosthetics, biomechanics, biological heat-transfer, biomaterials, etc.— which are technically developed out of precise requirements or needs highly specific to a certain application. Notwithstanding the inordinate efforts and advancements in wound reparation, investigation, and technologically developed products, the management of chronic and diabetic wounds still has shortcomings such as protracted infections, incessant inflammation, and decreased vasculo-angiogenesis. Besides, an injured dermis is the bed for microbial growth and proliferation leading to loss of vascular and other underlying tissues. Hence, we focus here on the contemporary wound healing bioengineered alternative agents, mainly nanoceuticals in the form of nanoparticles, scaffolds, and composites that, to a significant extent, have been able to assist wound healing.

In this review, we enumerate the pathophysiological, etiological, prognosis, and molecular mechanisms of wound healing. Additionally, we throw light on the traditional and contemporary therapies for healing and the current bioengineered alternatives being explored in the pharmaceutical technology field as part of wound management targeting in order to assist health specialists in overcoming the existing insinuations on wound valuation.

## 2. Wound Healing Management

### 2.1. The Process of Wound Reparation and Its Cellular Crosstalk Underneath

Wound healing upon acute damage begins with thrombogenesis with the cramming of immune cells and platelets that permeate the injury site to release several chemokines, cytokines, and growth factors [18]. Inflammatory phagocytes recruited to the wound site cause a steep rise in the cytokines and inducing inflammation [5,14]. They participate in the healing process by exuding bioactive molecules responsible for clotting, fibrous tissue formation, ECM deposition, vasculogenesis, epithelialization, and contraction [14,15,18]. Monocytes and macrophages also play a role in inflammation and tissue repair by initiating cellular proliferation and regulation.

#### 2.1.1. Inflammatory Cellular Response and Its Transition from Inflammation to Re-Epithelialization and Refurbishment

Whether it is skin, soft tissue, bone, or any organ, the response to damage/trauma is not unsimilar. A time-modulated and well-organized refurbishment of the dermis/epidermis tissue barrier is seen in acute wounds, unlike the unfinished repair with a predominant scar or a keloid formation in chronic ones [30]. The inflammatory and proliferative phases commence within 24 to 48 h post-damage, with the penetration of neutrophils followed by macrophages (cresting approximately until 5 days later), fibroblasts (7–9 days), and lymphocytes (cresting approximately on day 7) into the site of the wound. Hemostasis is attained by thrombocyte aggregation, vasoconstriction, and coagulation. The wound bed becomes the interim wound matrix framework for diverse cellular players channeling platelet degranulation and the complement pathway’s activation to stimulate inflammation [31,32]. Along with the hemostatic/coagulation phase, the inflammatory phase is designated as the ‘early phase of healing’, where the innate immune response is activated [33]. When the body senses an injury, the typical dermal cells, viz., keratinocytes, fibroblasts, dendritic cells, monocytes, and macrophages, are subjected to specific molecular pattern ‘threat’ signals from the host cellular stress responses or the pathogenic moieties like bacterial polysaccharides [34]. The immune response functions through pattern recognition receptors (toll-like receptors or TRLs), which smartly identify these ‘threat’ signals and activate the nuclear factor kappa-light-chain-enhancer of activated B cells (NF-κB). NF-κB is a crucial transcription factor in immune response, apoptosis, and inflammation, regulating various cytokines and chemokines and disseminating the inflammatory cellular response [33,34]. Several other immune-triggering key players specific to each phase drive the whole repair process and are described later, as and when they come into the ‘healing scene’.

Typically, with acute wounds, the inflammatory phase spans for the first few days and terminates when the cellular response stimuli have subsided. The innate and adaptive immune cell responses persist to function during all the repair stages [30,31]. As the inflammation subsides, the proliferative phase sets where new tissues comprising the collagen and other ECM components are restored (re-epithelialization) with simultaneous wound contraction through a well-knit vascular network and granulation tissue formation. Re-epithelialization is one of the most crucial phases during any epithelial or dermal wound repair and occurs immediately after the wound induction when the wound-edge keratinocytes start migrating. Basal keratinocytes rapidly migrate to the wound after 48 h post-injury. This migration is triggered by the aid of specific cell adhesion assemblies, namely, the desmosomal and hemi-desmosomal membranes that activate the calcium-dependent kinases, which in turn reorganize the cytoskeleton to drive migration [35]. Before their migration to initiate wound repair, keratinocytes embrace new wound-specific cell fibrin-rich matrices and alter their normal cell–matrix adhesions. The switching on/off of the regime of several integrins is crucial for the process of cell migration. For example, in mice models, the keratinocyte-specific knockout of b1-integrins can cause severe impedance in the re-epithelialization phase [36]. The enzymes collagenase, elastase, and hyaluronidase, also determining the cutaneous healing potential.

Blood vessel repair is also a crucial step in tissue restoration. Repair signaling molecules, such as nitric oxide, epidermal growth factor (EGF), keratinocyte growth factor (KGF), insulin-like growth factor-1 (IGF-1), and nerve growth factor (NGF) [37], stimulate the process of re-epithelialization. Vascular endothelial growth factor (VEGF), platelet-derived growth factor (PDGF), basic fibroblast growth factor (bFGF), and thrombin [38] begin the process of angiogenesis by activating the endothelial cells (storehouse of proteolytic enzymes). These enzymes dissolve the ECM basement membrane and allow the endothelial cells to seep out of the existing damaged vessels to form new blood vessel networks [39]. The proliferative phase consists of granulation tissue formation from the interim wound bed. The granulation tissue comprises a pool of fibroblasts, white blood cells, phagocytic cells, blood vessel networks, and collagen bundles that recuperate the damaged tissue’s structural and functional integrity [40]. Fibroblasts exhibit a significant role in preserving the skin’s homeostatic balance and orchestrating granulation tissue formation. Upon its migration into the interim wound bed, the activated fibroblasts secrete matrix metalloproteinases (MMPs) for simultaneous degradation of the wound matrix [40] and remodeling of ECM, ensuring wound closure. Myofibroblasts are capable of cell adhesion, binds, sequestrate, and initiating the repair process [41].

Clinically, the maturation phase commences after the development of granulation tissue, where myofibroblasts are driven by transforming growth factor beta (TGF- β) for the ready expression of α-smooth muscle actin (SMA) and the contraction of the wound [42]. Collagen III (a major component of ECM) is replaced by Collagen I with higher tissue strength. Myofibroblasts undergo caspase-mediated cell death upon the completion of remodeling. A decline in the new blood vessel network is observed, and a mature avascular environment is formed [30]. On the basis of the wound’s depth, the wound recovery is estimated; hair follicles and sweat glands detest complete recovery after severe trauma with only 3/4^th^ of the original structure and strength of the tissue achieved [30]. Irrespective of the wound site, there is a simultaneous synthesis and degradation of the extracellular matrix and MMPs that preserve the restoring tissue structure. The first line of defense is the stimulation of epithelial cells adjacent to the wound, which helps repopulate the cut edges and initiate the regulation of numerous genes around the edges [43]. Mouse transcriptome analysis experiments have revealed pieces of evidence. The early prime genes include the activator protein 1 (AP-1), a heterodimeric transcription factor of the Jun and Fos family protein moieties, along with Cys2His2 zinc-finger transcription factor Krox-26 [44]. The upregulation of target genes results in the proliferation and migration of keratinocytes [45]. Likewise, modifications are also necessary for cell–cell junctions. Malfunctioning of the junctions might lead to delayed healing; like the desmosome-keratinocyte junctions that acquire calcium dependency instead of being serine/threonine kinase-dependent contribute to unsteady cell adhesions and ultimately to late wound repair [15]. The MMPs come into the ‘foreground’ to link the integrin collagen as the epidermal barrier gets restored [43].

#### 2.1.2. Crosstalk of Keratinocytes and Fibroblasts during Healing

**Figure 2:**
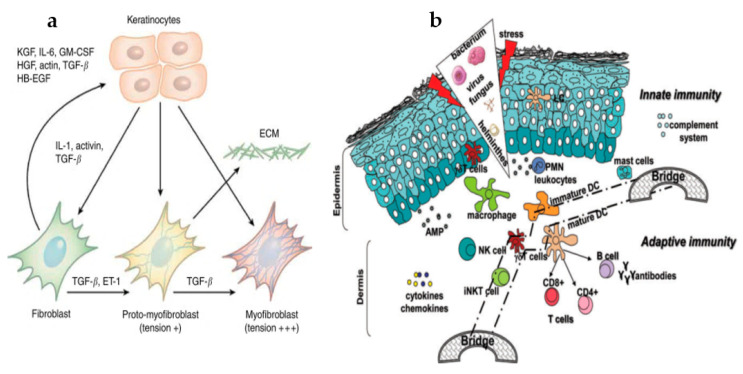
(**a**) Crosstalk between keratinocyte fibroblasts and fibroblast differentiation during wound healing. (**b**) Schematic illustration of the overall innate and adaptive immune responses in the dermal/epidermal surfaces (adapted from references [47,49], respectively, open access).

Mesenchymal–epithelial crosstalk, mediated by autocrine/paracrine regulatory mechanisms, initiates keratinocyte–fibroblast interaction, development, and differentiation, controls the MMP expression, and therefore, helps achieve skin homeostasis [40,45]. Keratinocyte sheets cultured in a keratinocyte-conditioned medium can accelerate wound epithelization and healing when used for transient wound covering in vitro [40]. In both wound repair and re-epithelialization processes, the keratinocytes and fibroblasts play a vital role. The mutual communication between the epithelium and mesenchyme for keratinocyte stem cell differentiation has been long established [45], and growth factors are the essential hook for epidermal proliferation. The keratinocyte seeded mesenchymal feeder cell cultures direct the fibroblasts to initiate the secretion of KGF/fibroblast growth factor-7 (FGF7), interleukin-6 (IL-6), and granulocyte-macrophage colony-stimulating factor (GM-CSF) [46]. Modulation of fibroblast proliferation and paracrine-mediated extracellular matrix formation in keratinocyte-conditioned medium demonstrated an upsurged fibroblast replication and reduced collagen formation during the repair process. Figure 2a gives a fair diagrammatic representation of keratinocyte–fibroblast crosstalk during the healing process [47]. Interruption in the keratinocyte–fibroblast coordination might also alter dermal fibroblast function. Postponements in the process of epithelialization escalate the incidence of fibrotic conditions. When keratinocytes cover the wound, only 22% of the structural site coordinated injuries develop fibrosis within the first few weeks, which reaches 78% when re-epithelialization happens after 21 days. Thus, it is understood that when epithelization is non-occurring, the extracellular matrix continues its deposition until a paracrine signal from the epidermal cells to the fibroblasts is received to slow down the healing of the wound [48].

#### 2.1.3. Crosstalk of Innate and Adaptive Immunological Response during Healing

Cellular crosstalk, synthesis, and secretion of growth factors, cytokines, chemokines, etc., are hallmarks of both nonspecific (innate) and immune effector (adaptive) cells that control the re-epithelialization and repair process. Innate and adaptive immune responses share a concurrent relationship, and currently, experimental indications for their use as novel therapeutics are being explored [49]. Similarly, it is necessary to throw light on the mechanistic approaches that keratinocytes and immune cells adapt for successful healing, which can very well be a new treatment option. Nonspecific immunity is the initial line of defense that exhibits instantaneous action in response to any trauma and eliminates host infection chances. This nonspecific cell reaction depends on certain molecular pattern structures highly conserved in microorganisms and is termed the pattern recognition receptors (PRRs) [50]. Both pathogen-associated molecular patterns (PAMPs) and damage-associated molecular patterns (DAMPs) participate in healing. While PAMPs are bacterial survival structures like bacterial endo/exotoxins, double-stranded deoxyribonucleic acid (DNA), and murein, DAMPs belong to cytoplasmic and nuclear protein components (high mobility group box 1 [HMGB1] proteins, heat shock proteins [HSPs], S100-β homodimer proteins, and purine metabolism) [51,52], released during the cell stress response, necrosis, acute inflammation, apoptosis, etc. Figure 2b represents the mechanism of innate and adaptive responses during the healing process.

PRR’s are solely expressed in antigen-presenting professional and non-professional cells and categorized into four major classes of TLRs, namely, C-type lectin receptors, retinoic acid-inducible gene-I-like receptors, and NOD-like receptors. TLRs are single-pass membrane-spanning receptors majorly expressed on the sentinel cells and are among the extensively studied PRRs. Upon activation, TLRs consequently also stimulate the NF-κB and MAPK cellular pathways with the help of a cascade of adaptor protein-signaling molecules: the myeloid differentiation factor 88 (MyD88) and MyD88 adaptor-like protein (MAL/toll-interleukin 1 receptor domain-containing adapter protein (TIRAP)), and the domain-containing adapter-inducing interferon-β (TRIF)-related adaptor molecules, which further activate and produce cytokines (interleukins: IL-1, IL-6, IL-8, IL-12) and tumor necrosis factor- α (TNF-α) [47,53]. Cytokines make sure that other small chemotactic cytokine molecules are triggered from the adjacent cells, which causes the migration of inflammatory cells to the site of injury to help the innate immune response set in [54]. Maturation of antigen-presenting accessory cells also occurs via the TLR-activated inflammatory mediator cells that bring about T-cell maturation and T-helper type 1 (Th1) polarization, thus employing the acquired immune response to come into the play and initiate the process of wound repair [54]. Adenosine A2A receptors, secreted in all human cells, can modulate to diminish inflammation and protect the tissues from inflammatory impairment. The A2A receptor controls the TLR-mediated cytokines and chemokines to be formed to accelerate wound closure. Clinical shreds of evidence have suggested that such adenosine receptor agonists enhance wound re-epithelialization and contraction in MyD88+/+ mice [55,56]. The acquired immune response, unlike an innate response, possesses an immunological memory that responds to any immunologic trial very quickly and is prolonged. However, a link between the two immunological responses and preliminary experimental establishments has shown that both these immunities co-exist and confer to wound healing [44,47]. Even though specific cells activate, functionalize, and link both these immune responses like the plasmacytoid dendritic cells, gamma delta T lymphocytes, and Langerhans cells, which are also the prime participants in wound healing, it is necessary to explore these mechanisms further for a clearer picture.

## 3. Interplay Amid the Key Players Involved and Their Effect in Deferred Wound Repair

A fair comprehension of the underlying cellular and molecular mechanisms and the interplay among various key factors associated with healing is vital. Moreover, the effect of these factors on non-healing intractable wounds is also something that cannot be unkempt. This section throws light on the role of key players in timely and untimely healing.

(*a*) *Scavenger white blood phagocytic cells—the macrophages*: Macrophages exhibit diversification in their functional phenotypes and retort differently to varying micro-environs of wound repair. The scavenging white blood cells are the only prime players who ‘work on’ all the repair phases [57]. Under normal skin conditions, macrophages are involved in maintaining a hemopoietic and homeostatic equilibrium within the system. Post-injury, monocytes accumulate around the site of the wound, and macrophage cells with an altered phenotype are concurrently activated and influenced by PRR moieties and natural killer cell-derived interferon-gamma. These then differentiate to M1 (classically activated macrophages), which release nitric oxide to curb intracellular pathogens, stabilize the host cell, and promote antitumor T-helper cells producing immune response [58,59]. The M2 (alternatively activated macrophages) set, possessing anti-inflammation, glucose regulating, and healing activity, is driven by the interleukin family (mainly IL-4 and IL-13) [60,61]. TLRs team up with immunoglobulin G (IgG) complexes to stimulate the macrophage cells to produce immunosuppressive IL-10 and TGF-β1 [59]. During hemostasis, the M1 type initiates phagocytosis, apoptosis, foraging of the cell remains, and induces interleukin proinflammatory mediators and TNF-α to trigger the leukocytic cells [62]. As the process progresses towards the inflammatory and proliferation phases, M1 is transitioned to the M2 set of cells producing decoy/regulatory receptors for agonist ligands of the IL-1 family and growth factors that promote fibroblast differentiation, ECM remodeling, and formation of new blood vessels [59]. Thus, the M1/M2 changeover is highly significant for assuming the persistence of the inflammatory phase and preserving the sense of balance to tissue restoration [60,62]. In the case of complex non-healing wounds, the functional modulation of the M1/M2 macrophage subset is fragmented. With chronic wounds, disruption in the M1/M2 phase may be due to iron overload within the macrophages that pushes them into an uncontrolled proinflammatory M1 activation state, which has been observed in venous ulcers and delayed skin repair [62]. Improper epithelization and delayed wound healing can occur during the later phases of injury by accumulating advanced glycation end products (AGEs) that trigger the macrophages to secrete unwarranted levels of TNF-α [60]. Transitory change of phagocytic cells from proinflammation to healing-allied phenotypes is the key for complete wound repair.

(*b*) *Endothelial cells*: Endothelial cells (EC), platelets, and the enzymatic breakdown of fibrin in blood clots are some of the prime factors that control hemostasis. Endothelial cells are the indirect reservoir of blood supply to the newly formed cells and tissues, supporting its development and subsistence and regulating inflammatory reactions in the cells [2,14]. The crucial processes, such as clotting, the regulation of blood flow, and the transport of plasma proteins into the tissues are functions of resting endothelial cells, which impede inflammation. Endothelium activation is essential in the process of inflammation during the repair. Endothelium undergoes two types of activation: In type I, the guanine nucleotide-binding protein (G protein)-facilitated receptors trigger G-protein αq subunits and instruct the cells to augment blood flow and plasma proteins into the tissue, endorsing the stimulation and binding of neutrophils, which finally erupts into the site of inflammation. In type II, TNF and IL-1 facilitate the augmentation of blood flow and help in the permeability of plasma proteins and simultaneous recruitment of leucocytes. Type-II-activated endothelial cells also function in recruiting neutrophil-mediated monocytes and T-helper cells for inflammatory reactions. In deferred wound healing, higher glucose or AGE levels could make the cells suffer higher apoptotic cell death, upregulated secretion of intracellular adhesion molecules, a cluster of differentiation (CD54 and CD106), increased production of reactive oxygen species and malonaldehyde, and lower levels of dismutase. This, as a result, activates the mitogen-activated protein kinase (MAPK) and NF-κB pathways and recruits the congregation of leukocytes onto the injury site [5,11,14]. The hyperglycemic environment then induces reactive oxygen species (ROS) production in the endothelial cells and in the bloodstream to restrict specific vasodilators—nitric oxide (NO) and prostaglandin I2—and upsurge certain vasoconstrictors—preproendothelin-1 (PPET1) and thromboxane—which initiate an inflammatory reaction to promote white blood cell adhesion and trigger TNF-α secretion to cause a deferment in wound repair [11].

(*c*) *Neutrophils, fibroblasts, and the keratinocytes*: The Neutrophils (a type of granulocytes), are the primary shields of innate immunity and respond to host infection or harmful mediators. These ‘suicidal killers’ adopt one of the three approaches to subside wound injury-mediated inflammation and initiate tissue repair. Firstly, neutrophils act as specialized phagocytes, removing tissue debris at the injury site. Secondly, mature neutrophils activate growth and pro-angiogenic factors to regenerate and revascularize the wounded tissue directly. Thirdly, neutrophils undergo apoptosis and are cleared up by macrophages [14,15] by a feed-forward mechanism of releasing tissue-repairing cytokines to accelerate tissue renovation. Neutrophils primarily act as decontaminators during the normal repair process. Still, an abnormalcy in their numbers in and around the wound site over time may contribute to the pathogenesis of non-healing wounds, as seen in patients with high blood glucose levels. Neutrophil serine proteases can damage ECM and specific essential repair proteins, e.g., clotting factors, complement systems, cytokines, and immunoglobulins [2], and build up oxidative stress in the cell [14,18]. Reports have shown that neutrophils are susceptible to apoptosis in hyperglycemic patients. A decrease in the neutrophil longevity and fast clearance from the infection site may lead to a prolonged infection phase. An in vitro study on diabetic rat models depicted the abundance of AGEs in skin tissues that hindered the binding of neutrophils to the surface receptors and triggered several cytokines to induce oxidative stress. This cytokine triggering and ROS production, in turn, affected the healing time [18]. 

Fibroblasts, or the structure stromal cells, are prime active regulators of wound healing and proinflammatory events [63,64]. These cells match up with the local stomal environment to regulate the level and kinetics of inflammation by interacting with the infiltrating inflammatory cells via CD40 receptors to activate the NF-κB complex and direct the fibroblasts to control the infiltration and function of immune cells by stimulating IL-6, IL-8, and cyclooxygenase-2 [64]. Inflammatory cells undergo apoptosis when cytokine production becomes deficit, and the inflammation is brought down [64]. Fibroblasts participate in the regulation of apoptosis with the aid of type I interferons (IFNs) [63]. Overall, fibroblasts do affect the inflammatory-proliferative phase transition by playing the ‘repair’ and ‘removal’ roles. However, chronic wounds compel fibroblasts to exhibit altered functionalities. In vitro experiments by Wang et al. [63] on the proliferation of fibroblasts indicated apoptotic cell death and deterioration in the proliferation of fibroblasts in the presence of certain glycation end products. A dose-reliant drop in fibroblast proliferation, collagen, and hyaluronic acid secretion with anomalous cytokine and matrix metalloproteinase expressions was also observed in a glucose-rich AGE medium. The fibroblast-mediated vascular endothelial growth factor (VEGF) remains impaired under hypoxic environments, and MMP-9 is overexpressed and triggers pro-degradative activity in diabetic mice model studies [65]. Diabetic fibroblasts fail to produce nitric oxide that is responsible for higher levels of MMP-8 and 9.

Keratinocytes, players of the proliferation phase of healing, do so by secreting proteins to rebuild the basement membrane and cause re-epithelialization. However, this regulation process goes haywire when a few unwarranted factors play in. A significantly higher NF-κB regulation of inflammatory response in keratinocytes was observed in diabetic rats in a study conducted by Takao and coworkers [65]. It is studied that the keratinocytes undergo an inverse concentration-dependent activation via AGEs, and higher concentrations of AGE inhibit keratinocyte proliferation by blocking the changeover from S to G2/M cell phase and by inhibiting the NF-κB signaling pathway and promoting the apoptosis of keratinocytes. More significant neural deposition of AGEs might increase cytoskeletal proteins, which can impair plasma transport to influence intracellular signaling and phosphorylation, ultimately leading to the degradation of axons [65]. The overaccumulation of AGEs causes the nerve vessel to constrict and concurrently associate with a signal transduction receptor on the endothelial cells to lessen iNOS production and blood flow, resulting in dysfunction of the nerves [65].

(*d*) *Biofilms*: Biofilms act as a blockade to the repair machinery. Continued studies have elucidated that biofilms ditch the normal curative path associated with ‘hard to heal’ chronic wounds undergoing deferred and abnormal healing with severe infection [66]. Chronic and acute wounds are vulnerable to such infections when there is a sterile loss in the inherent skin barrier function, enabling microbial entities to latch on and form biofilms within the wound milieu. As per clinical relevance, exposed wounds lack a protective sheath and house a variety of endogenous and exogenous flora of microorganisms that can be controlled during the preliminary healing phases either by internal immune defense or external suppositories. Nonetheless, a prolonged open wound bed can invite opportunistic pathogens to take over and proliferate and develop into the extracellular polysaccharide matrix or biofilms that are resistant to the immune system or antimicrobials [67]. An exposed wound with deprived blood circulation, necrotic debris, and hypoxic environs hinders the body’s innate immune defense system and provides a suitable attachment/growth medium for these microbes. Usually, chronic wounds are mired at the inflammatory phase of wound repair and exhibit elevated proinflammatory cytokines, proteases, and neutrophils; this, combined with microbial biofilm infection, further stalls the wound cure and disrupts its anatomical and functional integrity. The most frequently studied biofilm-forming pathogens in acute and chronic wounds are *Pseudomonas aeruginosa* and *Staphylococcus aureus* [67]. This is where investigations on the complexity and biochemical mechanisms of biofilms are still in the nascent stage and require thorough clinical studies to understand the exact pathogenesis and molecular mechanisms of biofilm-mediated wound infections; most of the initial research on apprehending such conditions on cells are evidenced by in vitro and in vivo models. The contribution of biofilms to deferred chronic wound healing has been elucidated with a few experimental data here. Experiments conducted on diabetic mice models with *Pseudomonas aeruginosa* biofilms revealed delayed wound contraction, raised blood sugar levels, and a 10-fold rise in IL-1β, IL-6, and MMP-10 gene expressions, even in the fourth week post-wounding when compared to control groups, thus depicting a biofilm-mediated protracted inflammatory response and repressed proliferation. The biofilm infection on wounds also showed thickened epidermal lining, poor vasculogenesis, and late re-epithelialization [68]. Roche et al. [69] studied the wound closure rates from 7 to 14 days post-wounding in methicillin-resistant *Staphylococcus aureus* (MRSA) biofilm covered dermal wounds in pig models. A significant delay in the closure rates with high bacterial load was observed in biofilm-affected wounds compared to control animals. A fibronectin receptor expressed by biofilm-forming Staphylococci does hinder the re-epithelialization by blocking keratinocyte migration via the matrix to the wound site [70]. In vitro models also play an imperative role in understanding the cell–bacterial interaction and overall multifaceted biofilm-mediated wound healing process. A comparative study on *Pseudomonas aeruginosa* and planktonic bacterial biofilms that affected granulocytes showed the *Pseudomonas aeruginosa* caused a decline granulocyte cell action and induced its apoptosis. Further study on rhamnolipids by Jensen et al. [71] presented that *Pseudomonas* producing rhamnolipids could induce rapid necrosis to granulocyte cells. A presumption is that the biofilm suppresses granulocyte cell activity and guards the bacterial cell against phagocytosis. In addition, Gram-negative bacterial lipopolysaccharides can persuade neutrophils to produce various chemokines and simultaneously alter the membrane-associated phosphatidylserine moieties to block the function of neutrophils [72]. It is evident here that multiple bacterial cell entities present in biofilms can sturdily distress several cellular processes in the inflammatory phase of healing. Though numerous primary in vitro and in vivo experimental results indicate biofilm pathogenesis, a highly intricate and specific unaided bacterial model is lacking to put on an accurate composite clinical representation without the utilization of human models.

## 4. Prospective Technologies of Wound Healing

### 4.1. Conventional Therapies Implemented for Healing

#### 4.1.1. Skin Grafting Techniques

Tissue grafting has been explored for a long time now, with the initial use of autografts going back as far as the 6th century. Skin grafts come into play when the tissue loss or injury is chronic. The graft thickness could either be split-thickness or full-thickness skin grafts [73]. Typically, split-thickness grafts use the epidermis and the papillary dermis of healthy adult skin for repair [73]. Split-thickness grating is known to be the gold standard for a variety of cutaneous wounds, but it comes with certain limitations. Split-thickness procedures fail to repair if the skin loss is more than 1/3^rd^ of the total area of body skin [73]. While meshing can increase the graft sites’ surface area, balancing the meshing ratio, which ideally should be no more than 3:1 (graft: wound area), is hard as it is prone to contracting during repair [74]. Post-grafting symptoms of ache, redness, and inflammation are also observed in such skin grafts. Contrary to split-thickness grafts, full-thickness grafts use both the epidermal and complete dermal layers and are advantageous in repairing soft tissue defects. A full-thickness skin graft can handle chronic injuries well, with less skin shrinking and more aesthetically natural-looking post-healing, unlike split-thickness ones [75]. Full-thickness grafts, however, need a fully vascularized bed for grafting and are affected by donor skin unavailability [76]. Lately, autologous skin graft efficiency has been improved by combining it with scaffolds, gels, therapeutic agents, etc., to accomplish massive full-thickness injuries [75]. Allotransplantation or homografts obtained from genetically dissimilar members of the same species are often beneficial in traumatic wounds, where a temporary graft covering alleviates the recipient’s wound bed until autografting is done [75]. Homografts are immediately available, increase donor supply, and extended storage before use, thus giving them an upper hand in the grafting method. Regrettably, allotransplants are often subjected to viral contaminations such as human immunodeficiency virus, cytomegalovirus, and hepatitis [76]. They might induce strong recipient inflammatory immune reactions with the interference of T and B lymphocytes to ultimately reject the homograft [76]. Recent experiments have shown that implementing mixed chimeric molecules with the donor’s bone marrow could subdue recipient graft rejection in clinical therapies such as the in vitro assay on RA-iTreg cells (retinoic acid), which exhibits immunosuppressive T-cell proliferative activity and also prevents T-cell cytokine activity in mice models [77]. On the other hand, xenotransplants are obtained from heterologous species, with the most frequently used being porcine xenografts, which are ready for use but can cause secondary infections from other dissimilar species. Usually used with burn wounds where <25% of the total skin area is affected, xenografts reduce the implementation of surgical excisions and save time [78].

#### 4.1.2. Wound Dressings

A dressing is considered ideal if it confers complete wound shielding, eliminates excess exudate, possesses antimicrobial efficacy, maintains a balance between optimum hydration and oxygen, is easy to handle, and has non-anaphylactic properties [24]. Most of the dressings have problems of frequent changing, contamination from the wound fluid, imbalance of wound moisture, difficulty to remove post-application, and insufficient antimicrobial protection. These problems were overcome by using cotton and polymeric bandages to treat dry wounds and those with mild exudation, like the nonocclusive dressing Xeroform ™, made up of a petroleum-based fine mesh gauze with 3% of bismuth tribromophenate, which was used for treating preliminary exudating wounds. Fabric-based non-allergic dressings saturated with paraffin and olive oil, such as Bactigras, Jelonet, Paratulle, etc., were commercially used non-adherent and gamma-sterilized dressings suitable for a superficial clean wound. However, these traditional dressings failed to provide an occlusive hydrated wound healing environment, which paved the way for modern alternatives, viz., contemporary formulated dressings applied directly onto wounds in the form of semi-solid creams, ointments, lotions, and liniments, as well as smart gels, thin self-adherent bioactive incorporated films, etc. The contemporary cotton gauge dressings incorporate chitosan-silver-zinc oxide nanocomposites for efficient moisture retention and antibacterial efficiency [24,25]. During the late 20th century, human amniotic membranes were used for exudate and fluid-laden burn wound dressings. These, as a non-cellular medium for adherence to mesenchymal stem cells, served as a vital platform for skin equivalent development [79]. Although such dressings were advantageous in providing temporary pain relief, balancing the optimum hydration in wounds, and were time- and cost-effective, the chance of infection spread was high [79]. Among polysaccharide dressings, chitosan and chitin are the most explored ones for clinical therapeutics because of their nontoxicity, biocompatibility, high durability, antibacterial efficiency, and suitability to be applied onto open wounds [24]. Their limitations include low tensile strength and elasticity. Algal extract-impregnated dressings have good absorbency, are hemostatic, and antimicrobial; thus, they are useful in exuding wounds [80]. The use of chitosan-alginate amalgamated dressings could improve the mechanical strength and stabilize the dressing [80]. Hyaluronic acid, a linear polysaccharide incorporated into dressings, is compatible with burn, chronic, and surgical wounds [25]. It gives structural sustenance to enable nutrient diffusion, clears wound debris by their interaction with the CD44 molecules, and balances hyperhydration during new tissue regeneration [25]. Hyaluronic acid dressings activate keratinocytes to migrate and proliferate wound sites for ready repair [81]. However, they are highly dissolvable and have less residence time in vivo. Microbial cellulose biosynthesized from *Komagataeibacter xylinus*, using diverse carbon and nitrogen sources, can precisely be made into dressings for the prophylaxis of extremely chronic injuries that require recurrent dressing changes [24,25]. Unlike other phytocelluloses, microbial ones show substantial pliability, strength, biocompatibility, and good absorbency, but their antimicrobial action limits their medical applications. In addition, they could be improved to a certain extent by the incorporation of nanoparticles like zinc-oxide nanoparticles. Hydrocolloid-based dressings are occlusive dressings for pressure ulcers [82]. They maintain an optimum water and oxygen balance within the wounds, but fail to hold a large amount of exudate. Their frequent changing is necessary to evade the maceration of tissues [82]. Foam dressings are bilaminar structurally with a hydrophilic end with moderate exudate absorbency for wounds with exposed bone. Foam dressings can be rightly called the new substitute to conventional dressings to treat venous pressure ulcers in preventing hospital-acquired pressure ulcers in critically ill individuals. However, they do not yet have much adherence to wound bed and hence are not indorsed for heavy exudative wounds [83]. Adhesive transparent film dressings are suitably conglomerated with hydrogels that allow for optimum wound hydration, maintain skin integrity, and easily monitor the wounds [25]. Research to expand their antimicrobial effectiveness has led to their combination with chlorhexidine, which displays high adherence and declines catheter-related infection to improve vascularization.

A variety of wound repair products for acute and chronic wounds have been doing rounds in the commercial market. Marketed wound repair strategies are mainly either traditional ones, with the sole function of wound coverage, or contemporary ones that have much more to offer and are superior in maintaining an optimal hydration environ for healing [2]. Some of the distinctive commercialized contemporary wound healing products are outlined in Table 1, along with their advantages, disadvantages, and applications.

Thus, a suitable wound healing material would readily absorb exudate, efficaciously maintain optimal hydration in and around the wounds, and would also regulate the site-specific delivery of various therapeutic agents to augment healing.

#### 4.1.3. Natural, Phytochemical, and Antiseptic Therapies

Natural and plant-based products have been the traditional ancestral therapies used in skin wound care and management before the rise of pharmaceutical and clinical alternatives. For centuries, these products, because of their potent antimicrobial, anti-inflammatory, anti-analgesic, and cell-stimulating characteristics, were implemented as traditional medicine for acute and chronic wounds. Owing to the existing incidence of diabetes and severe cardiac and vascular implications, chronic wound interventions seek significant attention, making the use of natural therapies for healing applications of specific interest. Natural amalgams encompass a widespread assortment of substances, antioxidants, phenols, terpenes, flavones, and many more organic and inorganic constituents that act as specific targets in the healing process [87]. These constituents have been clinically tested for their efficiency through in vitro and in vivo models. Since wound repair is a complex cascade of biochemical events, it is of utmost importance to stimulate a reparation process without microbial infections. Hence, traditional therapeutic agents and plant-based natural products have shown exemplary outcomes. Further scientific investigation on the progress of various extraction and purification methods, their precise mechanism of action, safety, and quality control assessments, etc., is obligatory. Traditional therapies are cost complaint and beneficial for primary wound care and management. Still, inconsistency in their batch-to-batch results, sudden immunologic reactions, and adverse after-effects can restrict their implication in multidisciplinary wound management [87].

In antimicrobial wound management, topical antiseptics form the first line of a cure for primary wound infection and reduce the microbial bioburden. Antiseptics permeate into wound biofilms, curb bacteria’s growth, and target a broad spectrum of microbial communities, unlike selectively specific antibiotic medications. The most frequently used antiseptics are povidone-iodine, chlorhexidine, alcohol, acetate, hydrogen peroxide, boric acid, silver nitrate, and silver sulfadiazine [88]. Ethanol, isopropanol, and n-propanol are the most suitable agents for surface decontamination and skin asepsis. Their antibactericidal spectrum targets both Gram-positive and Gram-negative bacteria. Another antiseptic used for centuries is iodine and its compounds, which act as a topical agent for preoperative skin preparations [89]. Cadexomer iodine, a hydrophilic modified-starch polymer bead, has been explored for the sustained and controlled release for treating wound exudates [90]. Chlorhexidine, belonging to the class of biguanide, has been extensively investigated as a biocide antiseptic in dermal and oral products either as a 0.05% dilution for wound cleansing or a 4% solution for surgical skin preparation and hand scrub [91]. Polyhexanide biguanide hydrogels have been explored for their cytotoxicity against methicillin-resistant Staphylococcus aureus on dermal wounds and their ability to clinically eradicate the infection. Bisphenols, viz., triclosan and hexachlorophene, are skin-compatible antiseptics effective against Gram-positives. Similarly, silver compounds in the form of solutions, creams, and ointments or nanocrystalline silver have long been used as antimicrobial agents. Their mechanical action is either through cell membrane lysis, the disruption of cellular protein and electron transport chains, or DNA levels by blocking transcription initiation [92]. The expediency of antiseptics on the skin surface, though they are fairly well-established and used, and their utilization as a prophylactic anti-infective agent for open wounds, remains a debatable and unexplored area in the present time. It is relevant to state that a combined traditional and modern therapy approach can target repair faster with negligible side effects, such as silver-impregnated nanofibers, aloe vera extract-embedded alginate hydrogels, propolis wound dressings, honey-based post-operative bandages, etc., could likely expand modern medicine.

#### 4.1.4. Mechanical Adjuncts and Physical Agents

Despite several attempts to equilibrate the cellular, biomolecular events during wound repair and preserve an optimal hydrated healing environment, there are times when wounds become chronic non-healing sites. A series of mechanical adjuncts and physical agents in use does contribute to such wound reparation processes and provide constructive and adjunctive functions. Hydrotherapy, ultraviolet C radiation (UV-C), vacuum-assisted closure, hyperbaric oxygen, and electrical stimulation are a few worth naming [93]. Hydrotherapy, being one of the oldest adjuvant therapies, is effective for burn wounds where a continuous rotation of water and air eliminates debris and toxic components and dilutes microbial colonization [94]. Hydrotherapy is advantageous for individuals with venous stasis dermatitis, pyoderma gangrenosum, peripheral artery disease, teeth lacerations, and rarely, diabetes mellitus acute wounds. This method effectively upholds optimal moisture in and around the wound surface for better revascularization and dermal regeneration. With several advantages, there are also a few disadvantages. A particular pressure of the water circulation is needed at the wound surface for rinsing of granulation tissue, which might impair the developing granulation tissue, restrict epidermal cell migration, and cause skin maceration [94]. In addition, bacterial infections can emerge if the moisture circulation is prolonged and proper drying of the wound is not done. Pulsed lavage therapy has currently become a replacement for hydrotherapy in terms of its use of an irrigating solution maintained at a particular pressure by a powered device [94]. The therapy improves the rate of granulation and better remodeling of wounded tissues. Ultraviolet C radiation (UV-C) ranges from 200–280 nm, and erythemal effectivity is accomplished at wavelengths of 250 nm where nucleic acid absorption happens, leading to accelerated DNA synthesis in fibroblasts, increased oxygenation and capillary blood flow for granulation tissue formation, and antibacterial and antiviral effects on wound surfaces. UV radiations can contribute to wound healing by upsurging epithelial cell turnover and hyperplasia to release prostaglandins and initiate cell proliferation for re-epithelialization. A dose-dependent application of these radiations may also cause shedding of peri-ulcer epidermal cells and sloughing of necrotic tissues and eschar [95]. Vacuum-assisted wound closure is applied to seal the wound area and place a negative pressure onto the wound surface that produces adhesive friction to the tissues and contracts wound depth for efficient closure [96]. This therapy can significantly and observably reduce water loss of the split-thickness graft area, curtail post-wounding duration, and restrict the relapse of infection during wound repair [96]. The accomplishment of vacuum-assisted closure therapy in treating chronic injuries has now led to its use in specialized clinical situations such as temporary abdominal closure, skin avulsion, poststernotomy mediastinitis, acute and subacute wounds, wound with bony prominence, osteomyelitis, as a graft reinforcement [96,97], and in reconstructive surgeries. Hyperbaric oxygen therapy confines a hundred percent oxygen at one atmospheric pressure to enhance oxygen inundation in the blood by forming oxyhemoglobin. Hyperoxic environments endorse wound repair through an increase in growth factors and formation of iNOS that regulates collagen formation, wound contraction, and endothelial progenitor cell proliferation [93,98]. This therapy has been utilized in chronic and poorly healing wounds, acute wounds, and diabetic foot ulcers. A systematic assessment of the healing capacity of hyperbaric oxygen therapy in diabetic foot ulcer patients was found to be clearly superior in comparison to other surgical procedures. Electrical simulation gathers both positive and negative charged cells, viz., neutrophils, phagocytes, epidermal cells, and fibroblasts, onto the wounded area so that each of the cells performs their specific cellular activities of wound healing. The endogenic electric field plays an imperative role in wound healing largely by triggering protein synthesis and cell migration. Clinical investigations have confirmed that electrical stimulation with steady direct currents is advantageous in wound acceleration. Human fibroblasts cells subjected to high-voltage pulsed current stimulation (HVPCS) did intensify the healing rate of soft tissue wounds as per reports [99]. Both protein and DNA synthesis rates became higher by applying specific blends of HVPCS voltages and pulse rates. Besides, both cell migration and a significant increase in the TGF-1β levels were prominent near the wound area in response to an endogenic electrical field that promoted early contraction and collagen synthesis (electrotaxis: the directional migration of cells toward the anodic or cathodic electrode of an applied electrical field) [99]. Researcher Yung Shin Sun experimentally aimed at optimizing the direct current stimulation therapy for enhancing the progression of wound repair. Via the finite element method (FEM), he standardized the distribution of endogenous electric fields produced around the wound area under the influence of different electrode configurations, including sizes and positions and the total power dissipation within different skin layers [100]. Extracorporeal shock wave therapy (ESWT) has been clinically relevant in being the cost-complaint effective intraoperative treatment for quite a few orthopedic and traumatic applications, together with challenging soft tissue wounds. These low-energy pulse waves were first clinically explored to treat urinary calcinosis, which later was utilized for treating bone and tendon injuries. Recently, ESWT has been studied to healing soft tissue wound injuries like burns, ulcers, etc., where these waves not only accelerate tissue repair, but also stimulate neovascularization in the underlying damaged tissues [101]. Additionally, these therapeutic shock waves also recruit newer mesenchymal stem cells, stimulate cell proliferation and differentiation, possess anti-inflammatory and antimicrobial effects, and block the toxic sensory pain receptors [101]. The underlying principle for ESWT employs short-term momentary acoustic pulsations with a simultaneous rise and fall in peak pressure time. The electro-hydraulic shock wave is generated within a metal inclusion, reflecting the shock waves towards the therapeutic target. Shock waves are electrically or mechanically produced energy waves that can traverse through a liquid or a gas medium. These waves either occur through electromagnetic, piezoelectric, or electrohydraulic wave pulses released from a high-voltage electrode water vaporization system [102]. During an injury, hypoxically challenged immunocompromised tissues are also subjected to ischemia. The application of ESWT onto the tissues increases tissue perfusion and reduces necrosis, as seen in experimental iatrogenic ischemia conditions [101]. Clinical research on experimental transgenic mice models displayed stimulation and upregulation of VEGF receptor 2, a mediator of angiogenesis, during ESWT treatments [103]. ESWT treatments also showed higher vessel densities in ischemic tissue formation experiments observed in immunohistopathological sections [104]. Another form of physical medication that uses low-level lasers or light-emitting diodes onto the body surface, known as the low-level laser therapy (LLLT), which has been effectively used as a therapeutic system to repair deferred wounds. This photothermal method comprises a monochromatic and coherent light source that produces a healing effect when the emitted photons are absorbed onto the wavelength-sensitive injured tissues/cells and trigger a set of complex biochemical arrays, ultimately resulting in accelerated healing of chronic wounds [105]. Lasers also employ their applications in burns, amputation wounds, skin grafting, infected wounds, etc. [106]. Low-intensity irradiations work between 500 and 1100 nm with laser output powers between 10 and 90 mW at the therapeutic site, capable of mediating cell proliferation and enhancing cellular activity. Visible red light, having the longest wavelength, is frequently used in LLLT, and has been seen to trigger faster healing of tissues by irradiating the mitochondrial complex and plasmalemma of the damaged cells, where the absorbed photons activate a set of enzymatic reactions and cytochromes, and energy conversions happen to yield adenosine triphosphates that, in turn, regulate cell function, relieve inflammation and pain, and accelerate healing [106]. Reports on the growth inhibition of *Pseudomonas aeruginosa, Escherichia coli,* and *Staphylococcus aureus* upon irradiation with 1–20 J/cm^2^ at wavelengths of 630 nm were seen in preventing wound infections [105]. LLLT may also show substantial positive results in stimulation and co-adjuvanted therapies. A study conducted by Pessoa et al. to inspect the co-effect of LLLT with steroid cortisone did show a co-adjuvanted effect of LLLT on cortisone to not only speed up the tissue healing process, but also to stabilize the harmful effects of steroids on tissues [107]. Neat corticosteroid, neat 5-fluorouracil, a pulsed dye laser (585-nm), and the amalgamation of all the three showed a significant enhancement in keloidal and hypertrophic scars post-treatment. Pulsed dye laser enhanced the scar surface and improved long-term adverse hypopigmentation, telangiectasia, and skin atrophy, unlike the corticosteroid [108].

### 4.2. Engineered Metal Composites Implemented for Healing

The nanoceutical adjuvants are biological entities that use nanotechnology to enhance their properties and garner their usage in diverse nano-based regenerative medicine applications. Nanoceutical materials possess lucrative physicochemical characteristics, which makes them of particular interest in various biomedical applications. Though scientific evidence regarding safety or efficacy is still being explored, the number of commercially viable nanoceuticals has magnified to a large extent. The growth trend of nanoceutical products is expected to continue and facilitate personalized medicine, targeted therapies with reduced side effects, and artificial intelligence-aided patient monitoring. However, mega challenges persist, particularly regarding biodistribution, metabolism in organs, and excretion from the body. Nanoceutical materials comprise designed metal-based nanoparticles and biomaterials that offer an unmatched approach to accelerate wound repair and the tissue-remodeling process. Their dimensions and shape govern their specificity, biological efficacy, cellular response, penetrability, and targeted delivery to the site of injury. These are comparatively nontoxic and exhibit high antibacterial properties [109]. In addition, other nanoceutical counterparts, such as the nanospheres, nano-capsules, nano-emulsions, nanocarriers, nano-scaffolds/composites, and nano-colloids, could serve as materials for wound tissue regeneration. The main focus here is metal-based nanoparticles as nanoceutical adjuvants for healing, followed by a brief overview of the other types.

Nanoparticles, both metallic and non-metallic, principally aid in wound repair and management by either possessing inbuilt inherent features that assist wound contraction or as delivery vehicles/carriers for assisted therapy. The most extensively studied ones are silver, gold, and zinc nanoparticles, owing to their unique dimensional, functional, chemical, and biochemical properties [109]. Silver nanoparticles (AgNPs), the potential candidate of choice for wound repair, have a high surface:volume ratio, show excellent activities at low concentrations, and are superior to the traditional silver compounds that were formerly used. Neat AgNPs can regulate the release of anti-inflammatory cytokines that facilitate rapid non-hypertrophic scar-devoid wound contraction [109,110]. AgNPs can initiate proliferation and differentiation of keratinocytes to augment epidermal closure and re-epithelialization and antibacterial efficacy. Myofibroblast differentiation from normal fibroblasts to promote speedy tissue renewal is also facilitated by AgNPs [109]. However, reports on their toxicity at increased concentrations by Szmyd et al. has shown that keratinocyte feasibility, absorption, migration, and differentiation are affected via specific cell death initiating the stimulus of caspase 3 and 7, ultimately leading to DNA mutilation [111]. Therefore, it is recommended to use lower safe doses, together with antimicrobic preparations (in the form of heat, radiation, and natural and synthetic chemicals, viz., phytochemicals, antiseptics, antibiotics, polymers, etc.), to attain improved effectiveness. AgNPs in conglomeration with the polyketide antibiotic can significantly reduce the bacterial load in epidermal and deep dermal layers in a mice model and quicken healing [110,111]. Hence, the combination of nanoparticles with conventional antibacterial mediators or dressings can more competently be used to repair infected wounds. Micro-cellulose reinforced with AgNPs behaves as an antimicrobial coating for open wounds and has shown high antibacterial performance against Gram-negative pathogens [112]. Experimental evidence by Chakrabarti et al. also depicted that coated polyester-nylon dressings with AgNPs can prevent biofilm formation and bacterial colonization, while upholding a low toxicity profile [112]. AgNPs form sulfur bonds with the bacterial plasmalemma proteins or bind to enzymatic thiol moieties, resulting in respiratory chain reactions and cell death [111]. Furthermore, these nanoparticles can hinder DNA synthesis and curb bacterial multiplication in the wounds. Experimental observations by Lu et al. showed that the incorporation of silica into AgNPs could give rise to nontoxic mesoporous disulfide structures, which can very efficiently adhere to open wounds and have outstanding bacteriostatic activity [109]. Ag-infused veneers warrant quicker wound healing and evade microbial colonization on the wound site, as observed in an in vivo canine model [109]. Commercially available silver-impregnated dressings, Acticoat ™ nano-sized AgNPs (<15 nm size) are currently being explored for their reparative, anti-infective, and pain-lessening facets for early burn wounds and may be complaint in evading burn wound infections upon their amalgamation with silver sulphadiazine and chlorhexidine digluconate formulation [113].

**Figure 3:**
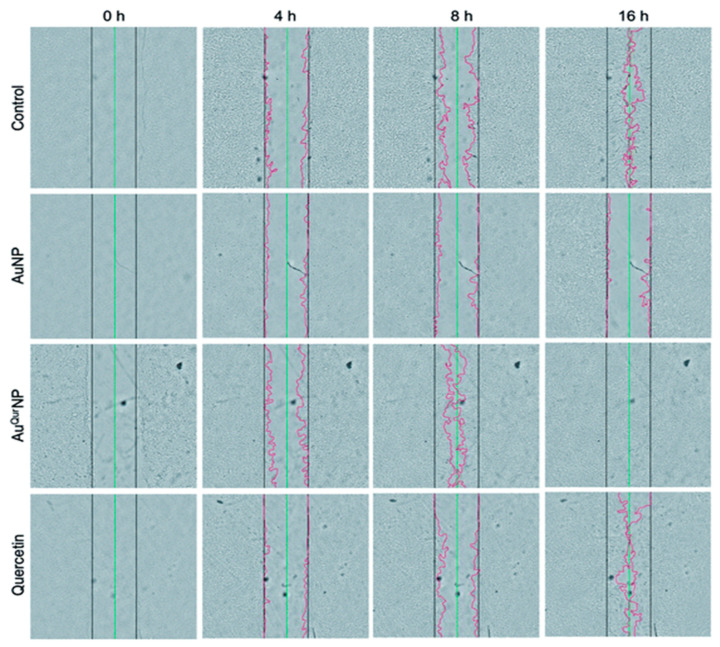
In vitro wound assay of human keratinocyte cells treated with AuNP (5 μg l^−1^), AuQurNP (5 μg l^−1^), or pure quercetin (15 ng ml^−1^) for different time periods (0, 4, 8, 16 h). Non-treated cells were used as control. Black, green, and red lines depict the start, end-point of cell migration, and migratory cell edge, respectively (magnification:10X). (reprinted with permission from reference [116]. Reproduced with permission of The Royal Society of Chemistry).

Gold nanoparticles (AuNPs) show potency in tissue rejuvenation, targeted drug delivery, and wound repair due to their extraordinary biocompatibility. AuNPs nanomaterials, because of their stabilizing properties, can be used as reinforcements with many other nanomaterials. Since neat gold particles do not exhibit a substantial activity, they need to be incorporated or combined with some matrix or therapeutic agent, a carrier, biomolecules, etc., for efficient antimicrobial activity. The cross-linking of AuNPs with collagen, chitosan, gelatin, and alginate, and their incorporation with various polysaccharides, growth factors, peptides, and cell adhesion proteins enables their attachment onto the gold nanoparticle surface without any alteration in the structural conformation of the biomolecule. These conglomerated moiety-modified AuNPs display excellent biocompatibility and biodegradability and are suited for healing. Similar to collagen, gelatin and chitosan can also easily be incorporated with AuNPs, showing safe and positive effects in enhancing wound healing [109,110]. Additionally, by modifying the surface plasmon resonance of AuNP, these exhibit thermo-responsive behavior, which is supported by in vitro and in vivo experimental data [109]. The mechanism of action of AuNPs follows either targeting the cell wall or binding to DNA to stall the double-helical structure from unwinding during replication or transcription, therefore contributing to bactericidal and bacteriostatic activities. They can thus show multidrug resistance to *Staphylococcus aureus* and *Pseudomonas aeruginosa*. AuNPs are also potent antioxidants [114]. Low concentrations of AuNPs are associated with keratinocyte growth and differentiation [109]. Observations made by Marza et al., on basic fibroblast growth factor AuNP-impregnated petroleum jelly mixtures showed enhanced angiogenesis and fibroblast proliferation, which aided speedy wound recovery [115]. The effect of colloidal AuNP coupled with quercetin (Au^Qur^NPs) on the fibroblast cell migration-assisted wound healing mechanism was depicted by Madhyastha et al. Au^Qur^NPs displayed enhanced cell proliferation and migration of keratinocytes, which was directed through the TGF-β1-dependent SMAD signaling pathway. This initial study on nanoceutical-engineered gold particles brings forth molecular and cellular evidence-based data to elevate the promising healing applications of Au^Qur^NPs in upcoming nanomedicine for skin etiology [116] (Figure 3).

**Figure 4:**
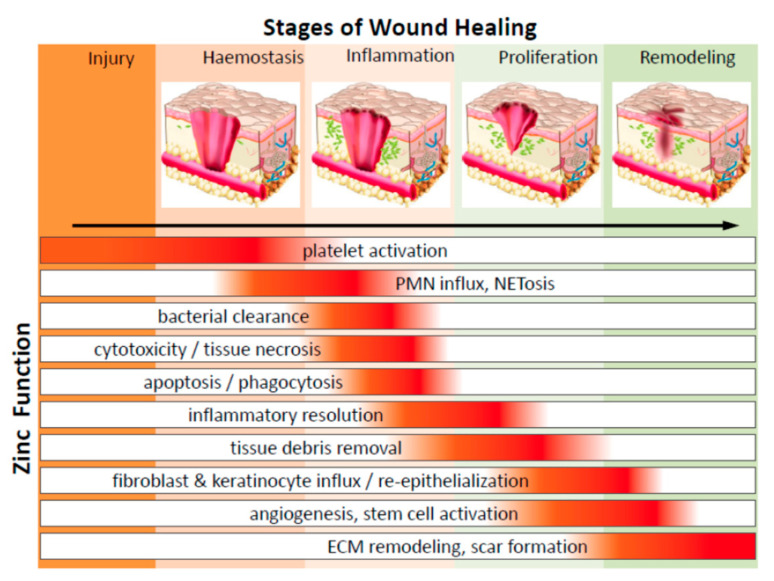
Diagrammatic representation of the significant function of zinc on cells in various phases of wound healing (PMN—polymorphonuclear leukocytes; NETosis—a novel form of programmed neutrophil death that resulted in the formation of neutrophil extracellular traps (NETs)) (adapted from Reference [118] respectively, open access).

Zinc-oxide nanoparticles (ZnONPs) exhibit potent antibacterial activity, and combined with hydrogel-based wound dressings [22], can activate keratinocyte migration and improve re-epithelialization [117] (Figure 4). A recent study on the assessment of ZnONP-based chitosan hydrogel formulations presented high absorbency of wound exudates and aided hemostatic blood clotting and antibacterial effectivity simultaneously [24]. ZnONPs and a collagen-based bioresorbable matrix with orange essential oil have been seen to substantially heal burn wounds, while also decreasing sepsis chances. This wound dressing was seen to augment angiogenesis, form new tissue, and exhibit biocompatibility and no cytotoxicity when evaluated in vitro and in vivo [118]. Yet, their inherent toxicity makes them less used in wound healing therapies [24]. ZnONP toxicity is dose-dependent, with higher doses it acts as a mitochondrial-dysfunctioning agent to release reactive oxygen species and block gene expression of superoxide dismutase and glutathione peroxidase in human keratinocytes, ultimately giving rise to oxidative stress and cell death. Creating core-shell nanocomposites by combining two metals, such as biogenic AuNPs with a thin coat of ZnO to form AuZnO core-shell nanocomposites, was assessed to evaluate the antibacterial and anti-biofilm efficacy against *Staphylococcus aureus* and methicillin-resistant *Staphylococcus haemolyticus* [119]. ZnONPs also have good tissue adhesive properties, as exhibited in mice skin models [109].

Nanoparticle-based composites: Renewable sources for nanoparticle synthesis and the development of its composites are gaining increased importance as they are cost-effective, consume less energy, and do not require additional sources for disposing of toxic by-products, unlike in nanoparticle synthesis via a chemical process. Several phytochemicals, viz., alkaloids, phenols, amino acids, proteins, etc., have been used to stabilize several nanoparticles like Ag ions in AgNPs. *Ocimum sanctum* merged AgNPs embedded into a Carbopol gel base and attained ≈96% of wound contraction by the 14th post-wounding day, as revealed by Sood and coworkers [120]. Additionally, they possessed activity against *Staphylococcus aureus* and *Pseudomonas aeruginosa*. Gelatin has in situ reductive properties to stabilize AgNPs, and the development of gelatin-chitosan-Ag porous composites with sizes 100–250µm that are biocompatible, biodegradable, and non-immunogenic, which upon cross-linking with tannic acid does exhibit therapeutic and antibacterial characteristics with low cytotoxicity [121]. Shao et al. [122] used *Barleria gibsoni* leaf extracts to synthesize ZnONPs gel formulation that showed substantial effect against Gram-positive and Gram-negative infected burn wounds. Polymers such as chitosan can also serve as nanomaterial dressings or drug carriers due to their biocompatible polymeric networks to hold optimum moisture for a balanced wound environment. Chitosan, being cationic, attract most metals, proteins, and dyes to form complexes [123], and its degradation products can activate ECM synthesis. Chitosan-assisted wound healing therapies include hydrogels, membranes, films, sponges, and scaffolds [123]. Chitosan nanoparticles have immunomodulatory and nontoxic effects on human dermal cells, as revealed by Chen et al. in his assembled acellular porcine dermal matrix using a naturally-derived chitosan oligosaccharide [124]. Additionally, the presence of the functional group aldehyde in AgNPs, formed during in situ reaction, confers the chitosan-AgNPs scaffolds with broad-spectrum antimicrobial properties against associated *Escherichia coli* and *Staphylococcus aureus* [125]. A combination of a chitosan-poly (vinyl alcohol) (PVA) complex improved the antioxidant and antimicrobial efficacy compared to the polymer alone. It also conferred strong Gram-negative activity against *Klebsiella* species and further enhanced in vivo wound repair by forming granulation tissue and re-epithelialization while demonstrating no cytotoxicity [125]. An infrared-irradiation-triggered thermo-sensitive hydrogel-based drug delivery system loaded with ciprofloxacin was developed by Gao et al., triggered by near-infrared light stimulation. The mixture of polydopamine nanoparticles/glycol chitosan, being photothermally active, generated hyperthermia leading to bacterial cell leaching. Besides, polydopamine nanoparticles in combination with the drug ciprofloxacin exhibit a controlled released when stimulated with near-infrared light and showed minimal leakage under physiological conditions [125]. Calreticulin (calcium-binding protein)-based AuNPs and chitosan/AuNP nanocomposites have been used for diabetic lesions. Calreticulins regulate the proper folding of proteins, and the nanocomposite endorses fibroblast-keratinocyte-endothelial cell growth, migration, division, and collagen formation without hindering cell proliferation [126]. The biopolymer cellulose triggers repair via multiple local growth factors such as the epidermal growth factor and basic fibroblast growth factor [127]. Nanocellulose dressings, due to their anti-infective properties and amplified tensile properties, have been explored as scaffolds [24]. Bacterial cellulose mimics the skin structure with a high surface area per unit, increased biocompatibility, hydrophilicity, and no cytotoxicity. 3D porous networks of nanocellulose have high water retention capacity, ensuring a moist environment appropriate for healing [24]. The wound healing potential of cellulose-ZnONPs composites was displayed by Khalid et al. [127]. Bacterial nanocellulose derived from Gram-negative *Gluconacetobacter xylinus*, and combined with silver nanoparticles, showed enhanced healing and reduced colonization of wound-associated *Staphylococcus aureus* in vitro [24].

Nanoparticle-embedded nano-scaffold systems: Nanoparticles in nano-scaffold systems for wound healing applications have escalated in the last few years. Nano-scaffolds, or better elaborated as nanofibrous scaffolds, are nano-systems premeditated to resemble the components of the cellular microenvironment or to reorientate cell behavior. The predicted tendency is to use biodegradable biomaterials, which help regenerate and repair damaged tissue [128]. With advanced tissue engineering tools such as nano-architectonics approaches, in which materials are designed taking into account methods including organic synthesis, self-assembly/self-organization, molecular manipulation, and structural regulation upon stimuli, scaffolds are being tailor-made to have good biodegradability, mechanical properties, and ease in processing some polymers to better fit into different tissue engineering arenas. At present, only a few techniques can successfully produce nano-scaffolds, and their consequent nanomaterials, within the nanoscale frame [129]. Electrospinning is among the few methods that results in uniform and stable morphological nanofibrous scaffolds [130]. The amalgamation of bioactives by direct dissolution into functional polymer solutions helps progress healing at different phases. Further, nanopolymers like the dendrimers also show anti-inflammatory and antibacterial characteristics. Studies on a porcine model of superficial partial-thickness wounds displayed enhance healing potency of an electrospun polymer nanofiber dressing with the least risk of infection. Chitosan-poly-vinyl alcohol nanofibrous scaffolds upon application to rat diabetic wound models had improved healing rates compared to controls [131]. An in vivo study in Wistar rats of a silver nanoparticle-spun nanofiber membrane exhibited numerous favorable effects of reduced cytotoxicity, broad-spectrum antibacterial action, abridged inflammation, and higher healing rates [132]. Recombinant human epidermal growth factor, another nanocarrier, has been revealed to stimulate healing of full-thickness diabetic wounds. Nevertheless, their restricted use is due to the highly proteolytic environment they possess and the downregulation of associated growth factor receptors and signaling molecules in the case of chronic wounds [133]. However, the results vary between experiments. Zhang et al. [134] defined a hydrogel with Ca^2+^ cross-linker as capable of releasing preloaded bFGF. Observations that both calcium and bFGF led to the growth and division of fibroblasts in the early re-epithelialization phases, persuading wound shrinkage on both in vitro and in vivo models. Nanofibrous mesh networks developed by electrospinning have been used for gene encapsulation in wound dressings. Gene-activated matrix therapy can simultaneously alter the expression of a target gene involved in regeneration and bridge the gap between tissue engineering and gene therapy. Wang et al. [135] optimized a gene delivery system based on the antimicrobial peptide LL-37 embedded on ultra-small AuNPs, which increased the complete antibacterial action in the topical treatment of diabetic lesions. Furthermore, a LL37-AuNPs composite boosted cellular and nucleus diffusion, thus accomplishing high gene delivery efficacy. This system possessed biocompatibility, endorsed angiogenesis through the expression of VEGF expression, and improved re-epithelialization and granulation tissue formation [136]. Nanoceria has scavenging activity due to the coexistence of two oxidation states (3+ and 4+) in the valence cerium atom. Hence, these nanoparticles may diminish oxidative stress and reinstate the balance between oxidants and antioxidant enzymes in diabetic lesions. A 100µg dose of cerium oxide nanoparticles-miR-146a combination enhanced diabetic wound healing without altering the wound tensile [136]. Stem cell therapy, another feather in the cap of tissue engineering and regenerative medicine, represents another possible beneficiary of the nano scaffold technology due to their substantial stem cell migration and differentiation. These multifaceted nanomaterials with numerous enhancing properties represent advantages compared to standard treatment procedures adopted in clinical practice.

## 5. Conclusions

Dermal wound healing has been extensively explored in recent times. It has encouraged dermal surgeons and technologists to inspect the fundamental underlying cellular and molecular mechanisms for developing innumerable therapeutic products for wound repair applications. A lacuna or scanty inkling on the therapeutic approach has efficaciously unraveled the difficulty of treating obstinate, sluggishly healing, or non-healing wounds. Nevertheless, a better awareness of features segregating chronic wounds from the non-chronic ones is essential for understanding the complete wound treatments. A complex cascade of events, such as bacterial inequities, increased incidence of inflammatory cells and proinflammatory growth factors, augmented protease synthesis in wound cells, drops in TIMP, are some of the implications affecting chronic wound healing. Improved understanding of these chronic wound features and their association with other factors must be well-thought-out when devising a novel therapy for increased treatment efficacy. Evaluation of several growth factors, their most effective amalgamations, their methods to control excessive inflammation and proteolytic forces, genetic therapy, development of skin counterparts, and the effectiveness of nanoceutical adjunctive treatments are essential key points and offer promising solutions to initiate proper healing especially in ‘hard to heal’ chronic and diabetic wounds. Additionally, translation of information and optimal delivery of chronic wound care is crucial along with understanding the apt patient outcomes. Hence, efforts of basic medical and advanced clinical research with interdisciplinary nanoengineering portmanteau are expected to offer a great potential for balancing the burden of chronic and diabetic wounds to a large extent.

## Figures and Tables

**Table 1:** Distinctive commercialized contemporary wound healing products.

Material Type	Marketed Names	Pros and Cons	Specific Applications
Polysaccharide (Alginate)	Algiderm Curasorb™ Algisite™	Quick absorption of exudates from wound bed and control of hemorrhage; Not suitable for dry and incessantly bleeding wounds	Primary and secondary infected wounds [80]
Hydrogel	Tegagel™ Transigel™ Hypergel® Nu-gel®	Provides easy absorption of wound fluid and exudates, comfortable and soothing to the dermis; The dermis adhesion and mechanical strength is poor	Irregular dermal surface wounds with mild exudates [84]
Hydrocolloids	Tegasorb™ Comfeel® Duoderm®	Excellent for granulation tissue development and accelerated wound contraction; poor breathability	Superficial burns and abrasive wounds [82]
Sponges	Polymem® Hydrasorb® Mepilex®	Easy wound fluid absorption capacity; no adhesion on dry wounds	Mild exudative wounds [85]
Transparent Films	Transeal® Tegaderm® Bioculsive®	Good elasticity and ductility; Unable to absorb exudates and difficult to remove	Appropriate for puncture wounds [86]

## Data Availability

Not applicable.

## Abbreviations

ECM	extracellular matrix
RNA’s	ribonucleic acids
TRL’s	toll-like receptors
NF-κB	nuclear factor kappa-light-chain-enhancer of activated B cells
EGF	epidermal growth factor
KGF	keratinocyte growth factor
IGF-1	Insulin-like growth factor-1
NGF	nerve growth factor
VEGF	vascular endothelial growth factor
PDGF	platelet-derived growth factor
bFGF	basic fibroblast growth factor
BM	bone marrow
MMPs	matrix metalloproteinases
TGF- β	transforming growth factor beta
SMA	α-smooth muscle actin
AP-1	activator protein-1
FGF7	fibroblast growth factor-7
IL-6	interlukin-6
GM-CSF	granulocyte-macrophage colony-stimulating factor
PRRs	pattern recognition receptors
PAMPs	pathogen-associated molecular patterns
DAMPs	damage-associated molecular patterns
DNA	deoxyribonucleic acid
HMGB1	high mobility group box 1
HSPs	heat shock proteins
MyD88	myeloid differentiation factor 88
MAL	MyD88 adaptor-like protein
TIRAP	Toll-interleukin 1 receptor domain-containing adapter protein
TRIF	Toll-interleukin 1 receptor domain-containing adapter-inducing interferon-β
TNF-α	tumor necrosis factor- α
Th1	T-helper type 1
cAMP	cyclic adenosine monophosphate
G-protein	guanine nucleotide-binding protein
CpG	cytosine triphosphate deoxynucleotide-phosphodiester-guanine triphosphate deoxynucleotide
Rho GTPases	Ras homologous guanidine triphosphatases
Rac1	Ras-related C3 botulinum toxin substrate 1
Wnt	Wingless-related integration
M1	classically activated macrophages
M2	alternatively activated macrophages
IgG	Immunoglobulin G
AGEs	advanced glycation end products
EC	Endothelial cells
CD54	Cluster of Differentiation 54
CD106	Cluster of Differentiation 106
MAPK	mitogen-activated protein kinase
ROS	reactive oxygen species
RAGE	Receptor of advanced glycation end products
PPET1	preproendothelin-1
NO	nitric oxide
VEGF	vascular endothelial growth factor
MRSA	methicillin-resistant *Staphylococcus aureus*
UV-C	ultraviolet C radiation
HVPCS	High-voltage pulsed current stimulation
FEM	finite element method
AgNPs	silver nanoparticles
AuNPs	gold nanoparticles
Au^Qur^NPs	Gold nanoparticles coupled with quercetin
ZnONPs	Zinc oxide nanoparticles
PVA	poly (vinyl alcohol)
miR-146a	microRNA-146a
IRAK-1	interleukin-1 receptor-associated kinase 1
TRAF-6	tumor necrosis factor receptor-associated factor 6
